# Mice Lacking Serotonin 2C Receptors Have increased Affective Responses to Aversive Stimuli

**DOI:** 10.1371/journal.pone.0142906

**Published:** 2015-12-02

**Authors:** Stephen J. Bonasera, A. Katrin Schenk, Evan J. Luxenberg, Xidao Wang, Allan Basbaum, Laurence H. Tecott

**Affiliations:** 1 Division of Geriatrics, Department of Medicine, University of California San Francisco, San Francisco, California, United States of America; 2 Department of Psychiatry, University of California San Francisco, San Francisco, California, United States of America; 3 Department of Anatomy, University of California San Francisco, San Francisco, California, United States of America; Kent State University, UNITED STATES

## Abstract

Although central serotonergic systems are known to influence responses to noxious stimuli, mechanisms underlying serotonergic modulation of pain responses are unclear. We proposed that serotonin 2C receptors (5-HT_2C_Rs), which are expressed within brain regions implicated in sensory and affective responses to pain, contribute to the serotonergic modulation of pain responses. In mice constitutively lacking 5-HT_2C_Rs (2CKO mice) we found normal baseline sensory responses to noxious thermal, mechanical and chemical stimuli. In contrast, 2CKO mice exhibited a selective enhancement of affect-related ultrasonic afterdischarge vocalizations in response to footshock. Enhanced affect-related responses to noxious stimuli were also exhibited by 2CKO mice in a fear-sensitized startle assay. The extent to which a brief series of unconditioned footshocks produced enhancement of acoustic startle responses was markedly increased in 2CKO mice. As mesolimbic dopamine pathways influence affective responses to noxious stimuli, and these pathways are disinhibited in 2CKO mice, we examined the sensitivity of footshock-induced enhancement of startle to dopamine receptor blockade. Systemic administration of the dopamine D_2_/D_3_ receptor antagonist raclopride selectively reduced footshock-induced enhancement of startle without influencing baseline acoustic startle responses. We propose that 5-HT_2C_Rs regulate affective behavioral responses to unconditioned aversive stimuli through mechanisms involving the disinhibition of ascending dopaminergic pathways.

## Introduction

Although serotonergic neurotransmission has been implicated in the modulation of responses to noxious stimuli, the nature of these controls and their underlying mechanisms are unclear. Inconsistent results have been reported, and this likely relates to variability in dosing and selection of pharmacological agents, the modality of noxious stimuli studied, and the heterogeneity of serotonin receptors. At least 14 receptor subtypes mediate the actions of central serotonin [1,2], and the relative contributions of distinct 5-HT receptor subtypes to the sensory and affective components of pain perception remain to be clearly delineated.

Several lines of evidence indicate that 5-HT_2C_Rs contribute to the serotonergic modulation of nociceptive responses. 5-HT_2C_Rs are expressed in multiple brain regions implicated in nociception, including the bed nucleus of the stria terminalis, amygdala, prefrontal cortex, and striatum [3–7]. However, diverse and inconsistent results have been reported in studies of 5-HT_2C_R agonist and antagonist treatment effects on nociceptive processing. For example, antinociceptive effects of 5-HT_2C_R activation were indicated by the antiallodynic effects of 5-HT_2C_R agonists in different rodent pain models [8–11]. For example, pharmacological blockade of 5-HT_2C_Rs decreased the antinociception produced by nefopam (acetic acid induced writhing and formalin-induced paw licking; [12]), fluvoxamine (mechanical allodynia following sciatic nerve lesion; [13]), post-ictal state (heat-induced tail flick assay; [14]), intraperitoneal acetic acid conditioning (formalin-induced paw licking; [15]) and intrathecal serotonin (formalin-induced paw flinching; [16]). However, not all studies reported antinociceptive actions of 5-HT_2C_Rs. For example, 5-HT_2C_R agonist treatments enhanced nociceptive responses in rodents receiving intraperitoneal injection of acetic acid [17] or hindpaw injections of formalin [18]. Additionally, 5-HT_2C_R antagonist treatment was reported to suppress mechanical allodynia produced by sleep deprivation [19].

A well-known action of 5-HT_2C_Rs of potential relevance to their modulation of pain responses relates to the regulation of ascending brain dopamine pathways. Activation of 5-HT_2C_Rs located in the substantia nigra pars compacta and the ventral tegmental area has been found to suppress activity of nigrostriatal and mesolimbic dopamine pathways, respectively [20–23]. We demonstrated that 2CKO mice exhibit enhanced activity of both pathways [24–26]. In this context, it is noteworthy that ascending dopamine pathways are activated by noxious stimuli, and this activation has been associated with the magnitude of nociceptive responses in rodents and in humans [27–31]. In light of the above findings, we considered the possibility that disinhibition of dopamine pathways could contribute to the enhanced processing of nociceptive messages in animals lacking 5-HT_2C_Rs.

## Methods

### Mice

All studies were carried out in accordance with the NIH Guide for the Care and Use of Laboratory Animals. Procedures describing the generation of 2CKO mice have been previously described in detail [32]. Experimental cohorts were generated by breeding C57BL/6J^*htr2c+/htr2c-*^ females to wildtype C57BL/6J^*htr2c+/Y*^ males. All studies used 2–3 month old male wildtype and 2CKO littermate offspring. Animals were fully congenic to the C57BL/6J genetic background. Mice were group-housed and maintained under standard UCSF transgenic facility housing conditions. All experiments were conducted by investigators blinded to genotype.

### Baseline nociception assays

We determined baseline levels of sensitivity to noxious heat, mechanical and chemical stimuli in wildtype and 2CKO mice (for detailed procedures see [33]). Briefly, in the Hargreaves’ assay of sensitivity to noxious heat, animals were acclimated for 30–60 min to clear plastic chambers atop a glass surface through which a radiant heat source was focused on a hindpaw. The heat source was adjusted to produce a baseline response of approximately 10 sec in wildtype mice. For the hot plate test, animals were placed on the hot plate at temperatures of 48.0°C, 52.5°C, or 55.0°C and the latencies of mice to flinch, jump or lick their hindpaws was recorded. Hot plate temperatures and corresponding heat exposure cut-off times were as follows: 48.0°, 60 sec; 52.5°C, 60 sec; 55.0°C, 30 sec. Sensitivity to mechanical stimuli was assessed using calibrated von Frey filaments. Mice were placed in a clear plastic chamber with a wire mesh grid floor and hindpaws were stimulated with filaments applied through the mesh floor. Testing began using the 0.4 g von Frey hair. Withdrawal thresholds were determined using the up-down method [33,34]. To determine sensitivity to a noxious chemical stimulus we used the formalin test, in which mice received an intraplantar hindpaw injection of 2% formalin in a volume of 10 μl. Time spent licking/biting the injected hindpaw was assessed at 5 min intervals over a 60 min post-injection period. Twenty-four hours after the formalin test, we monitored sensitivity to mechanical stimuli using the von Frey test on the injected and uninjected hindpaw. A profound drop in mechanical threshold after formalin administration indicates mechanical hypersensitivity produced by chemical injury to peripheral nerve terminals [35,36].

### Ultrasonic vocalization

Ultrasonic vocalizations were recorded as described by Liu *et al* [37]. Mice were restrained in acrylic holding chambers and placed in a sound-insulated testing chamber. Brief unconditioned footshock stimuli of varying intensity (0.04 mA, 0.1 mA, 0.16 mA, 0.2 mA, 0.4 mA, 0.8 mA, 1.6 mA) were presented to the mice in a pseudorandom order; 70 stimuli were presented per trial, with average interstimulus intervals of 60 sec. Custom designed hardware was built to synchronize stimulus generation (using SRLAB, San Diego Instruments, Inc) with our MATLAB-based (The Mathworks, Natick, MA) sound data acquisition system. A microphone capable of measuring ultrasonic frequencies (Brüel & Kjær Falcon 1/4” condenser microphone 200 V Pol, free field, 4 Hz-100 kHz, type 4939) was fixed about 10 cm from the animal. The microphone signal was amplified (Brüel & Kjær Nexus conditioning amplifier), filtered (Tucker-Davis technologies antialiasing filter, F_c_ = 120 kHz), digitized (Tucker-Davis technologies A/D converter), and saved as a binary sound file (sampled at 250000 Hz) using custom-written MATLAB software. To compare wildtype and 2CKO sensory response to noxious stimuli, we measured the magnitude of vocalization-during-shock (VDS) responses (*i*.*e*., total sound and ultrasonic power during presentation of stimulus). To compare affect-related responses to noxious stimuli in wildtype and 2CKO mice, we determined the magnitude of vocalization-after-discharge (VAD) responses (*i*.*e*. total sound and ultrasonic power following termination of the shock stimulus). Both response latency [38] and spectrographic morphology [39] criteria were used to differentiate VDS from VAD responses. Briefly, VDSs were characterized by their temporal occurrence during the shock presentation, and by visual inspection of the resulting sound spectrograms, which demonstrated constant energy distribution across a continuum of audible and ultrasonic frequencies. In contrast, VAD responses occurred at variable latencies after termination of shock presentation; visual inspection of the resulting sound spectrograms demonstrated significant harmonic components in both audible and ultrasonic frequencies (“chatter” per [39]).

### Fear-sensitized startle

The fear-sensitized startle (FSS) assay was adapted from Kokkinidis and colleagues [40–42]. Mice were transferred from their home cage into individual transport cages, placed in a Plexiglas holding chamber within the startle apparatus (SRLAB, San Diego Instruments, Inc.), and exposed to a 5 min acclimation period in the darkened chamber with 70 dB white noise (dB measures obtained at sea level with 20 μPa as reference sound pressure). Acoustic startle responses were measured using a piezoelectric device that responded to the deformation of the holding chamber as the mouse startled. Baseline acoustic startle responses were obtained at stimulus magnitudes of 90, 105, and 120 dB (white noise; nine replicates for each stimulus magnitude; stimuli were presented in a pseudo-random order separated by 60 sec). The order of stimuli was as follows (from 1^st^ to 27^th^): 105 dB, 120 dB, 90 dB, 120 dB, 90 dB, 105 dB, 90 dB, 105 dB, 120 dB, 105 dB, 90 dB, 120 dB, 120 dB, 105 dB, 90 dB, 90 dB, 120 dB, 105 dB, 90 dB, 105 dB, 120 dB, 105 dB, 120 dB, 90 dB, 120 dB, 90 dB, 105 dB. Startle stimulus parameters were 0 ms rise time, 40 ms hold time, 0 ms fall time; startle response data were collected for 2000 ms starting 20 ms before stimulus presentation. Presentation of all pre-shock startle stimuli required approximately 30 min. Immediately after evaluation of pre-shock startle responses, mice received a series of nine 0.4 mA, 250 ms unconditioned shock stimuli, each separated by 60 sec. Following shock stimuli, startle responses were again measured in a manner identical to the pre-shock phase. Post-shock startle testing began 60 sec after presentation of the final shock, and required approximately 30 min to deliver all startle stimuli.

### Drugs

Raclopride (Sigma-Aldrich), a dopamine D_2_/D_3_ receptor antagonist, was dissolved in saline vehicle and administered at doses of 1 and 3 mg/kg (10 μl injected volume per gram body weight). Mice received *i*.*p*. injections of drug or vehicle 20 min before testing.

### Statistical analysis

Outcomes (spectral power in 22–100 kHz frequency bands for ultrasonic vocalizations, piezoelectric voltage response for startle) were compared by univariate analysis of variance (ANOVA). For startle experiments, we performed full factorial analyses evaluating primary factor effects (genotype, startle stimulus intensity, condition (pre- *vs*. post- shock responses, *aka* shock sensitization) and drug dosage where appropriate), as well as all factor interactions. When required, *post hoc* testing was performed by Student’s *t*-test of wildtype *vs*. mutant vocalization responses corrected for multiple comparisons by the Bonferroni method.

## Results

### 2CKO mice exhibit normal baseline sensitivity to noxious thermal, mechanical and chemical stimuli

**Fig 1:**
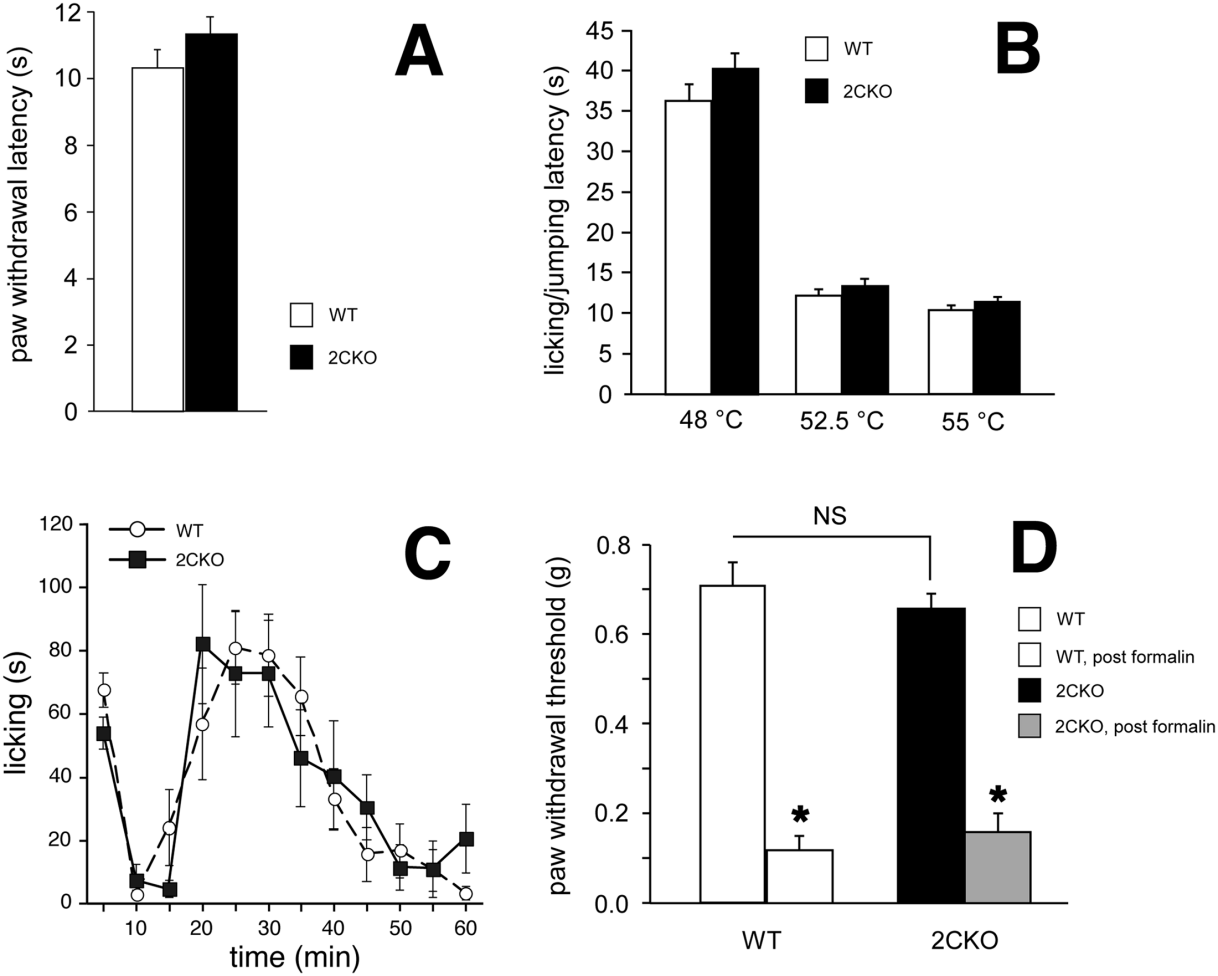
Heat-induced nociceptive responses are similar in 2CKO and wildtype mice. A. No difference between wildtype (open bar) and mutant (filled bar) paw withdrawal latencies in Hargreave’s assay. Data analyzed by Student’s *t*-test, bars are ± 1 standard error. B. No difference between wildtype (open bar) and mutant (filled bar) latencies to move paws from hot plate at 48, 52.5, and 55°C. C. No acute difference in the temporal pattern of paw licking following hindpaw formalin injection. Data analyzed by ANOVA, bars are ± 1 standard error. D. Twenty-four hours after formalin injection, paw withdrawal thresholds significantly decreased to a similar extent in 2CKO and wildtype mice. * = *p*<0.001, Students’s *t*-test, bars are ± 1 standard error.

In the Hargreaves’ test of heat nociception, paw withdrawal latencies to a thermal stimulus did not differ between wildtype and 2CKO mice (Fig 1A; Student’s *t*-test, *p* = 0.17). Similarly, there were no differences in licking/jumping latencies in the hot plate test (Fig 1B; Student’s *t*-test, *p* = 0.13, 0.21, 0.13 at 48.0°C, 52.5°C, and 55.0°C, respectively). The responsiveness in the formalin test was also comparable in the wildtype and 2CKO mice (Fig 1C; *p* = 0.96, F = 0.0023, two way repeated measures ANOVA). Importantly, not only were baseline mechanical withdrawal thresholds comparable in the two groups of mice, but the mechanical hypersensitivity recorded postformalin did not differ (von Frey test, Fig 1D). For each of these nociception assays, *n* = 10 for both wildtype and 2CKO mice. Taken together these results indicate that the loss of 5-HT_2C_Rs does not alter baseline responses to a diverse range of noxious stimuli and that nerve injury induced mechanical hypersensitivity is preserved.

### Selective phenotypic enhancement of affect-related vocalizations in response to footshock

**Fig 2:**
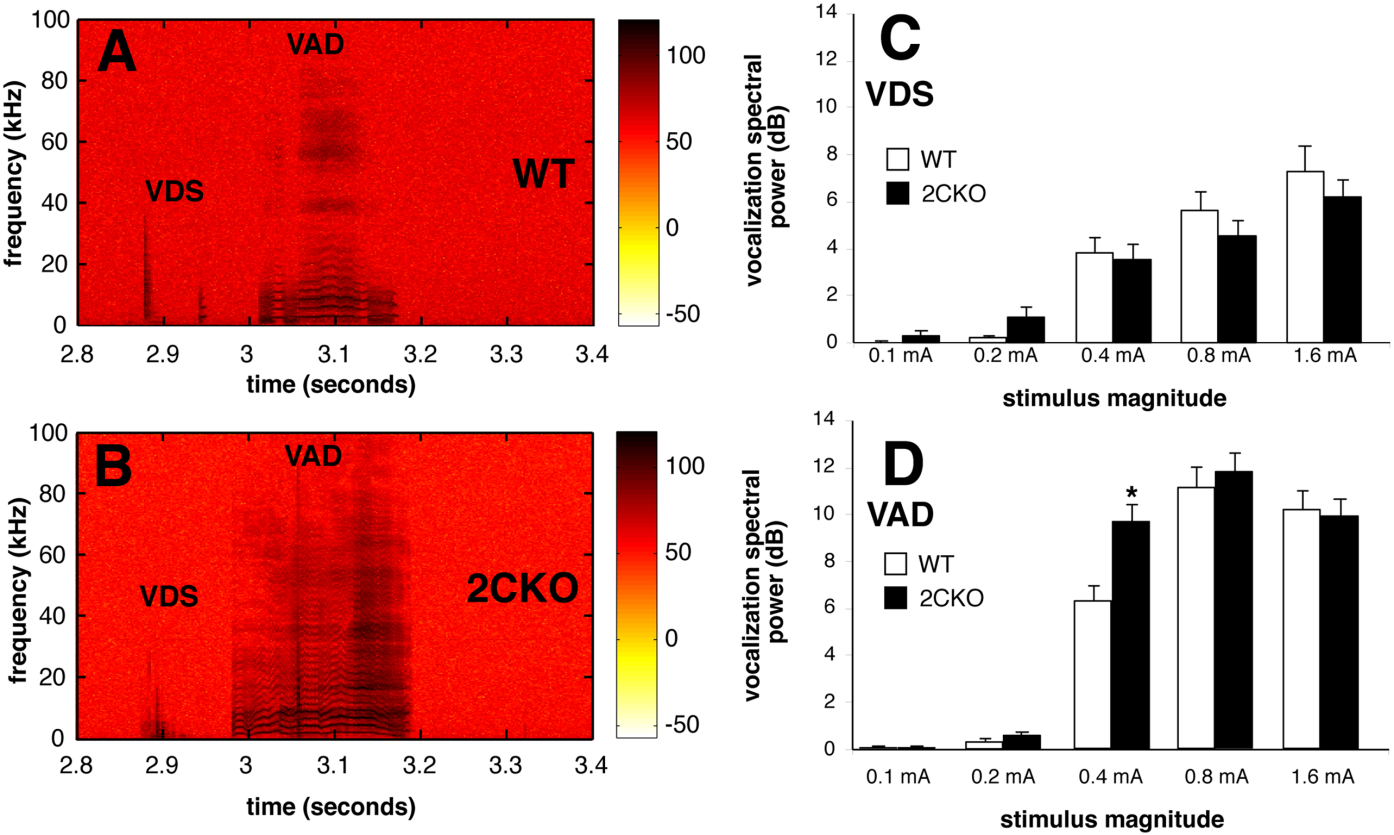
2CKO mice display ultrasonic vocalizations to footshock indicative of enhanced affective reactivity. A. Representative sound spectrogram from a wildtype mouse following a 0.4 mA footshock stimulus administered 2.7 sec into recording trial. Two vocalization-during-shock (VDSs, two short noise spikes) followed by vocalization-after-discharge (VAD, long noise burst with visible “chatter” lines) vocalization patterns can be observed. B. Representative sound spectrogram from a 2CKO mouse following a 0.4 mA footshock stimulus. A single VDS vocalization followed by a prolonged VAD (with prominent chatter) is observed. C. Spectral power (above ambient noise) of VDS vocalizations. Open bars depict wildtype responses, and filled bars depict 2CKO responses. Bars are ± 1 standard error. D. Spectral power (above ambient noise) of VAD vocalizations. Open bars depict wildtype responses, and filled bars depict 2CKO responses. * *p*<0.001, Student’s *t*-test, bars are ± 1 standard error. Since our background noise measurements averaged around 70 dB, the background ultrasound power for these figures shows up in red on the colormap, with higher powers depicted as darker colors.

Analysis of ultrasonic vocalization following shock stimuli is an established method of evaluating nociceptive responses in rodents [43–45]. Analysis of vocalization timing and spectrographic morphology has been proposed to distinguish sensory from affective responses to shock stimuli [38,39]. Here we recorded ultrasonic vocalizations in response to graded shock stimuli (ranging from 0.04 mA to 1.6 mA) were recorded in wildtype and 2CKO mice (*n* = 8 per genotype). Spectrographic analyses of vocalizations were performed to quantify total power in both audible (0–22 KHz) and ultrasonic (22–100 KHz) frequency ranges for both the vocalizations elicited during the shock stimulus (VDS) and the vocalization afterdischarge (VAD) occurring at variable latencies following shock stimulus termination. Fig 2A and 2B illustrates representative spectrographic traces showing the characteristic waveforms of both VDSs and VADs for wildtype and 2CKO mice.

Using standard sound spectrographic morphological criteria to distinguish VDS and VAD responses, we calculated total ultrasonic spectral power for all of the above responses. No significant phenotypic differences in VDS ultrasonic spectral power were noted. Increases in stimulus strength significantly increased VDS power to a similar extent in both groups (Fig 2C; *p*<0.03 for stimulus strength, genotype and stimulus x genotype interaction NS by ANOVA). These findings indicate that mice lacking 5-HT_2C_Rs display normal sensory responses to footshock. By contrast, at the 0.4 mA stimulus intensity, 2CKO mice exhibited a significant increase in VAD power (Fig 2D; *p*<0.001 for stimulus strength; *p*< 0.023 for genotype, *p*<0.011 for stimulus x genotype interaction by ANOVA; *post hoc* testing of 0.4 mA group by Bonferroni method, *p*<0.002). This is suggestive of a heightened affective response to the 0.4 mA stimulus.

### 2CKO mice exhibit enhanced fear-sensitized startle

**Fig 3:**
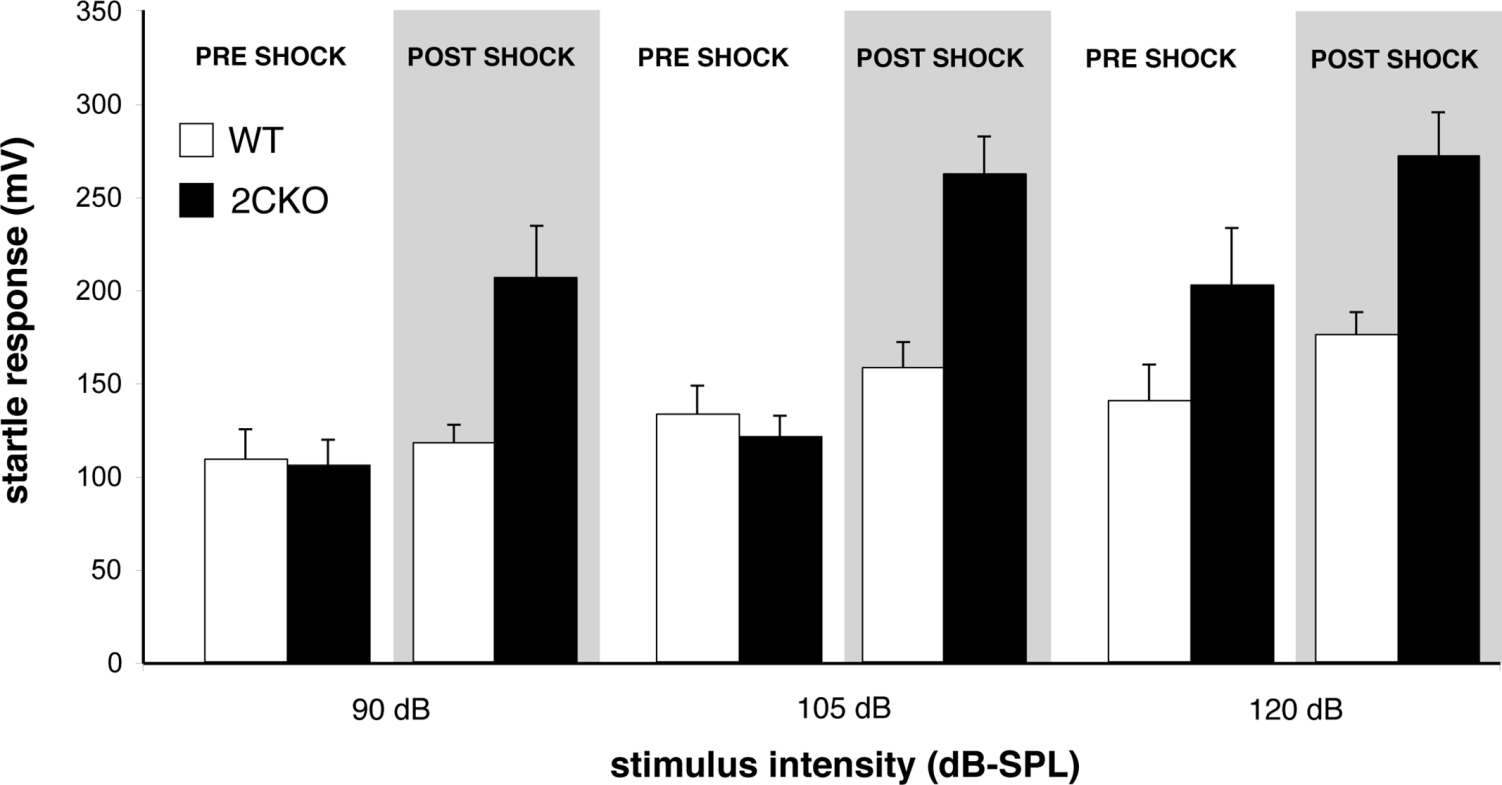
2CKO mice display enhanced fear-sensitized startle responses after unsignaled footshock. Open bars depict wildtype, and filled bars depict 2CKO data. All footshock stimuli were 0.4 mA in amplitude. The primary factors of genotype, startle stimulus intensity, and condition (pre *vs*. post shock) were all significant at *p*<0.001, ANOVA, bars are ± 1 standard error.

Fig 3 illustrates startle magnitudes before and after shock sensitization. Unless otherwise noted, the magnitude of the shock stimulus was 0.4 mA for 0.25 sec, which in mice is considered to be mildly noxious [46]. Whereas wildtype and 2CKO (*n* = 8, 12 respectively) mice had similar baseline startle magnitudes, the enhancement of startle following shock presentation was significantly greater in the mutants (Fig 3; by three-way ANOVA, *p*<0.001 for genotype (F = 31.46), shock sensitization (F = 24.35), startle stimulus intensity (F = 10.29, and genotype x shock sensitization interaction (F = 12.63), no other interaction terms significant).

**Fig 4:**
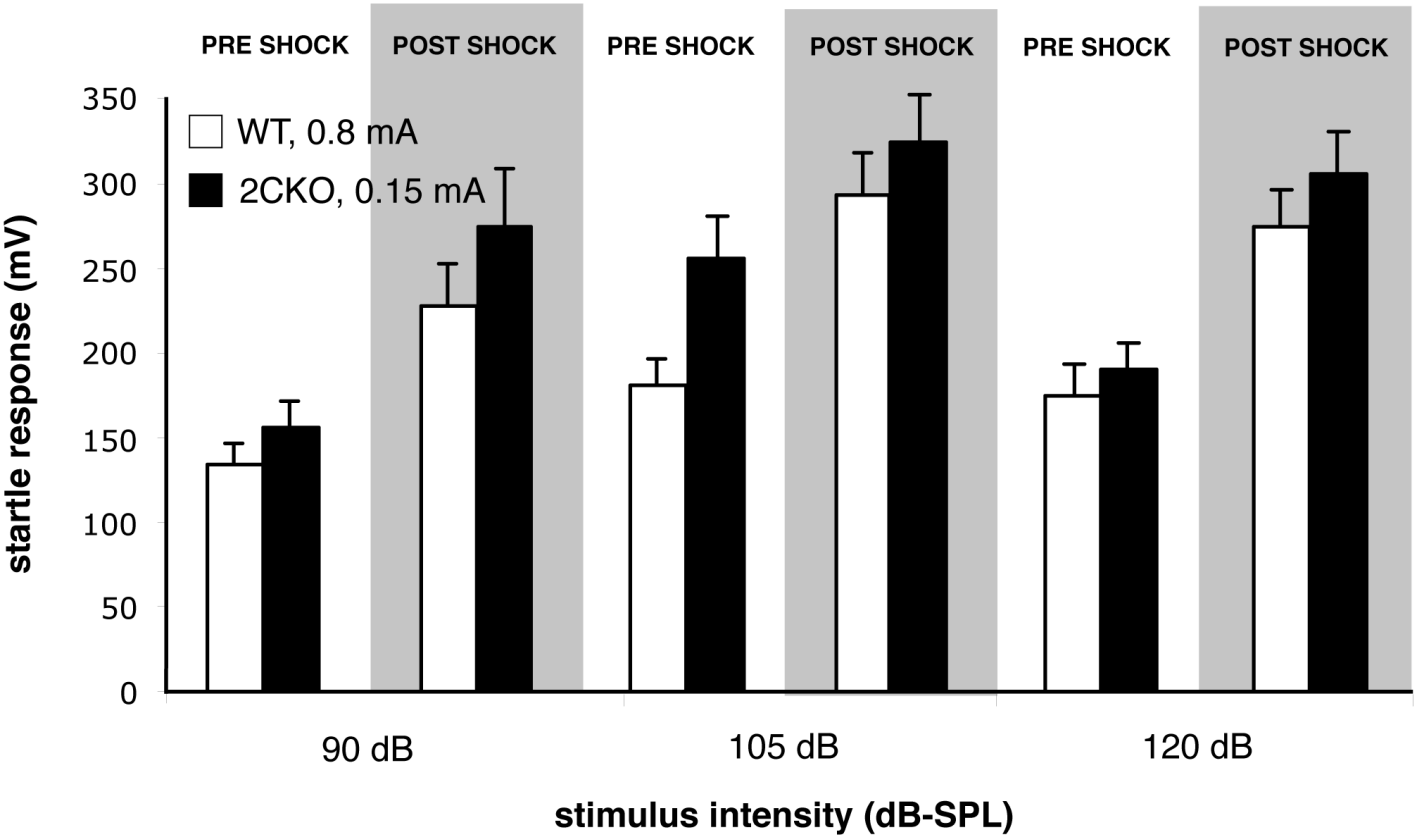
Wildtype mice require higher shock intensities to exhibit levels of startle potentiation equivalent to those of 2CKO mice. Responses of 2CKO mice to footshocks of 0.15 mA intensity (black bars) are similar to those of wildtype mice receiving 0.8 mA shocks (filled bars). ANOVA, bars are ± 1 standard error.

The greater sensitivity of 2CKO mice to shock-induced startle enhancement was further demonstrated by the observation that comparable startle responses resulted from different shock stimulus intensities in wildtype (*n* = 10) and 2CKO (*n* = 8) mice. Fig 4 shows that startle facilitation produced in wildtype mice by a shock intensity of 0.8 mA was equivalent to that produced by 0.15 mA in mutants. In this experiment, the primary factors of genotype, shock sensitization, and startle stimulus intensity were all significant by 3-way ANOVA (*p*<0.001, F = 7.393, F = 57.019, F = 7.768 for genotype, shock sensitization, and startle stimulus intensity respectively, no two-way or three-way interaction terms significant).

We note that on initial inspection, it could be difficult to understand why, for 2CKO mice, absolute post-shock startle values were greater in the study using the 0.15 mA shock (Fig 4) than in the study using a 0.4 mA shock (Fig 3). However, further examination revealed a nonspecific enhancement of startle responses in the former study, affecting not only post-shock 2CKO responses, but also pre-shock 2CKO and wildtype responses. When shock-induced enhancement of startle was examined as percentage increase relative to pre-shock values, the increased facilitation in 2CKO mice by the 0.4 mA stimulus was significantly greater than that resulting from the 0.15 mA stimulus (3-way ANOVA interaction between Study and Shock Condition; F = 261284; *p*<0.001).

### Selective block of fear-sensitized startle in 2CKO mice by the dopamine D_2_/D_3_ receptor antagonist, raclopride

**Fig 5:**
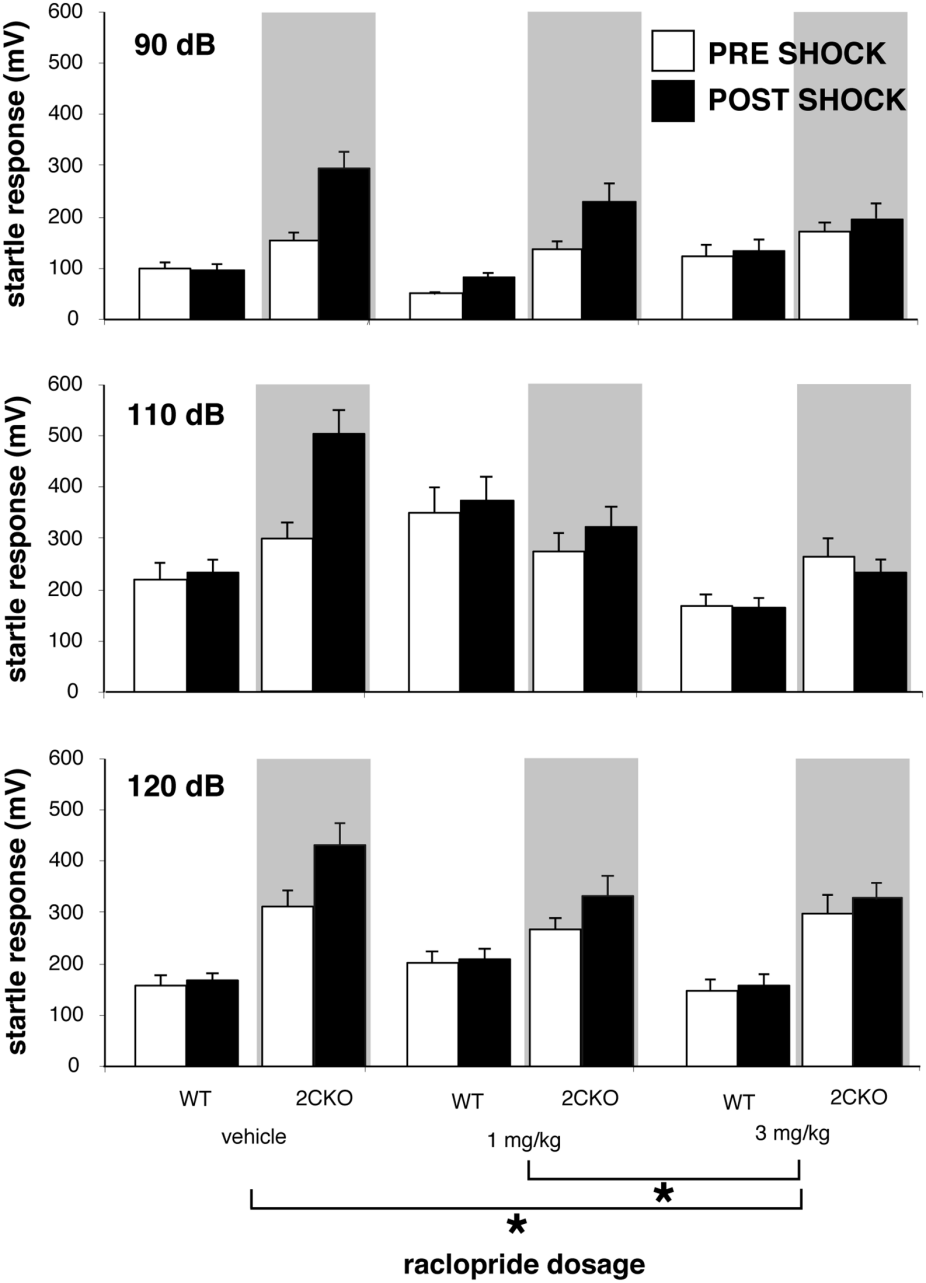
Raclopride blocks expression of fear-sensitized startle responses in 2CKO mice without affecting baseline startle responses. Open bars depict preshock responses, black bars depict postshock responses. Bars surrounded by no background derive from wildtype mice; bars surrounded by grey background derive from 2CKO mutant mice. Raclopride dosages given on x axis. A. Responses to 90 decibel (dB) startle stimuli. B. Responses to 105 dB startle stimuli. C. Responses to 120 dB startle stimuli. Whereas the footshock-induced enhancement of startle responses in 2CKO mice is blocked by raclopride, baseline startle responses are unaffected by the drug. ANOVA, bars are ± 1 standard error.

Previous studies of 2CKO mice revealed evidence for enhanced activation of mesolimbic dopamine pathways [25]. This is notable in light of prior studies demonstrating that FSS is sensitive to limbic dopamine D_2_ receptor activation [47]. We hypothesized that if enhanced mesolimbic dopamine system activation contributed to the observed phenotype, then administration of dopamine D_2_ receptor antagonists could eliminate the phenotypic differences that we recorded for fear-sensitized startle responses. We therefore examined such responses in wildtype and 2CKO mice following treatment with saline vehicle or the dopamine D_2_/D_3_ receptor antagonist raclopride. Fig 5 illustrates startle responses both before and after the shock stimulus in mice that received vehicle, raclopride 1 mg/kg, and raclopride 3 mg/kg (*n* = 8 per genotype/dosage combination). Four-way ANOVA with genotype, stimulus magnitude, drug dosage, shock as primary factors to model startle response demonstrated a significant genotype × condition × dosage interaction suggesting that increasing doses of raclopride suppressed FSS in mutant mice to a greater degree than in wildtypes (*p*<0.035, F = 4.437). Primary factors of mouse genotype, raclopride dosage, startle stimulus intensity, and pre *vs*. post shock condition were all significant predictors of startle response (*p*<0.001, F = 119, F = 10.8, F = 69.6, F = 13.1, respectively), as were 2 way interactions of genotype by raclopride dosage, genotype by startle stimulus intensity, genotype by pre vs. post shock condition, raclopride dosage by startle stimulus intensity, and raclopride dosage by pre *vs*. post shock condition (*p*<0.005, F = 13.6, F = 5.23, F = 17.4, F = 6.03, F = 7.76, respectively). Raclopride treatment of 2CKO mice normalized fear-sensitized startle responses in a dose-dependent manner, with an enhanced effect on 2CKO startle responses.

## Discussion

Our studies not only reveal an impact of 5-HT_2C_R function on the regulation of nociceptive behaviors, but also that the influence of these receptors appears to be selective for the affective response to noxious stimuli. Baseline withdrawal responses did not differ, which indicates that transmission of the “pain” message from the periphery to spinal cord reflex circuits was not altered. These findings indicate that serotonergic influences can differentially modulate sensory versus affective aspects of pain processing. To our knowledge this is the first report of an experimental manipulation that selectively enhances affective responses while sparing sensory responses to a noxious stimulus. The ability of a selective dopamine D_2_ */*D_3_ receptor antagonist to suppress the phenotypic enhancement of such affective responses raises the possibility that 5-HT_2C_Rs exert these effects through regulation of ascending dopaminergic pathways.

The preservation of the sensory component of pain processing was not limited to a particular modality. Thus no phenotypic differences were observed in paw withdrawal responses to either a thermal (Hargreaves’ test) or a mechanical stimulus (von Frey filaments). Furthermore, 2CKO mice displayed normal levels of formalin-induced paw licking, a behavior believed to reflect sensory rather than affective-motivational response to the chemical stimulus [48]. Consistent with this perspective, a prior study demonstrated that lesions of the anterior cingulate cortex, a region implicated in affective-motivational aspects of pain, did not impact these behavioral responses to formalin. On the other hand, the anterior cingulate cortex lesions did eliminate behaviors that are more associated with the affective-motivational response to formalin (conditioned place avoidance; [48]). Not only did the 2CKO mice exhibit normal acute responses to formalin injection, but there were also no phenotypic differences in the subsequent nerve injury-induced mechanical hypersensitivity of the injected paw, which is presumed to arise from nerve damage produced by formalin.

These results are consistent with our finding that 2CKO mice exhibited normal VDSs during electrical shock. VDSs occur with short latencies during shock stimuli, with spectral powers highly correlated with stimulus intensity [38,39]. In contrast, mutants displayed a phenotypic enhancement of VADs, which occur following shock termination, and exhibit a characteristic spectrographic pattern distinct from VDSs. Several features of VAD responses indicate that they are reliable correlates of nociception-induced affect [38,49–51]. For example, VAD responses are more sensitive than VDSs to suppression by anxiolytic drug treatments, or by damage to forebrain regions linked to affective pain responses in humans, and they are selectively enhanced by stressors [38,49,50]. Moreover, the capacity of electrical shock to support fear conditioning is directly related to its capacity to elicit VADs [52,53]. Taken together, the enhancement of VADs, but not VDSs in 2CKO mice suggests that there is a selective increase of the sensitivity of these animals to affective responses to noxious stimuli.

Consistent with a phenotypic enhancement of affect-related nociceptive responses, we found that 2CKO mice also displayed increased sensitivity in an entirely different test situation, one that is also believed to measure an affective response. The enhancement of the acoustic startle response following administration of electrical shock has been considered to reflect an affective pain response elicited in rodents [42,54,55] and in humans [56]. Baseline acoustic startle responses were normal in 2CKO mice, consistent with prior studies indicating that baseline auditory physiology is normal in these animals [57]. By contrast, relative to wildtype mice, mutants displayed markedly enhanced startle responses following footshock. The footshock intensity used in this procedure (0.4 mA), had been previously shown to elicit normal VDSs in mutants, indicating a lack of phenotypic influence on their sensory response to this stimulus. The enhanced responsiveness of 2CKO mice to footshock-induced startle sensitization was further highlighted by the observation that wildtype mice required footshock amplitudes of 0.8 mA to potentiate startle responses to the level observed for mutants receiving 0.15 mA stimuli. Altogether, the normal baseline nociceptive responses to a variety of noxious stimuli in 2CKO mice, coupled with evidence of enhanced responsiveness in two distinct assays of nociception-induced affect with very different behavioral output measures, indicate that genetic lesion of the *htr2c* locus selectively augments affective *vs*. sensory responses to noxious stimuli.

We considered the possibility that disinhibition of the mesolimbic dopamine system contributes to the augmentation of nociception-induced affective responses in mice lacking 5-HT_2C_Rs. The activity of mesolimbic dopamine pathways is subject to serotonergic inhibition, and pharmacological studies that used 5-HT_2C_R antagonists suggest that 5-HT_2C_Rs located within the ventral tegmental area contribute prominently to this action of serotonin [23,58,59]. Accordingly 2CKO were found to exhibit elevated levels of extracellular nucleus accumbens (NAc) dopamine, enhanced cocaine-induced dopamine efflux in the NAc, and enhancement of behaviors associated with mesolimbic dopamine function [24,25].

It is therefore of interest that mesolimbic dopamine pathways terminating in regions such as the NAc, amygdala and prefrontal cortex have been implicated in affective responses to noxious stimuli, including footshock [28,30,60,61]. For example, pharmacological blockade of dopamine D_2_ receptors in the amygdala suppressed the expression of fear responses in a Pavlovian conditioning procedure that used footshock [62]. Evidence for nociception-induced activation of the mesolimbic dopamine system in humans was reported in a positron emission tomography study using displacement of raclopride as an indicator of dopamine release [31]. The extent to which application of a noxious stimulus activated NAc dopamine release was highly correlated with ratings of negative affect and fear during noxious stimulus exposure.

We hypothesized that if disinhibition of dopamine pathways contribute to the enhancement of affective responses to noxious stimuli in 2CKO, then the phenotype could be attenuated by dopamine receptor blockade. Indeed we found that treatment with raclopride selectively blocked shock-induced sensitization of acoustic startle in mutant mice without affecting baseline startle responses. These findings are consistent with prior work demonstrating that shock-induced sensitization of acoustic startle could be suppressed by amygdala injections of raclopride [47]. Altogether, our findings indicate that the absence of 5-HT_2C_Rs selectively enhances affective responses to noxious stimuli via a dopamine D_2_ receptor sensitive mechanism.

Further studies are required to determine definitively whether this phenotype is attributable to disinhibited mesolimbic dopamine system activation, and the locations of the mesolimbic terminal fields most involved. Possibilities include the NAc, the amygdala and the prefrontal cortex. It is notable that in addition to the ventral tegmental area, 5-HT_2C_Rs are also expressed in each of these regions, where they could exert local actions impacting pain-associated affect. It should also be noted that 5-HT_2C_Rs are widely expressed throughout the brain, and could possibly influence pain responses via dopamine-independent mechanisms [3].

The findings reported here were performed in mice completely lacking 5-HT_2C_Rs throughout development. Therefore, we cannot exclude the possibility that developmental consequences of the 5-HT_2C_R loss could contribute to the phenotypes observed. However, a recent study indicates that lifelong global modifications of 5-HT_2C_R function can impact pain sensitivity in humans [63]. A positron emission tomography study using radiolabeled raclopride was performed with subjects bearing a common allelic variant of the *HTR2C* gene. In the Cys23Ser variant, a serine replaces a cysteine in the N terminus of the variant receptor, which has been reported to display reduced serotonin binding affinity [64–67]. Cys23Ser carriers exposed to a nociceptive challenge displayed higher overall qualitative experiences of pain, increased NAc dopamine release, and there was a significant correlations between these measures [63]. In this context, further analysis of the neural mechanisms through which 5-HT_2C_Rs influence affective responses to nociceptive stimuli, warrant consideration for exploiting serotonergic mechanisms in pain management and for understanding genetic predispositions to pain vulnerability.
